# ASK ME!—Routine measurement of patient experience with patient safety in ambulatory care: A mixed-mode survey

**DOI:** 10.1371/journal.pone.0259252

**Published:** 2021-12-01

**Authors:** Katja Stahl, Oliver Groene

**Affiliations:** Department Research & Innovation, OptiMedis AG, Hamburg, Germany; PLOS: Public Library of Science, UNITED KINGDOM

## Abstract

**Objective:**

Routine measurement of patient safety from the patients’ perspective receives increasing attention as an important component of safety measurement systems. The aim of this study was to examine patients’ experience with patient safety in ambulatory care and the results’ implications for routine patient safety measurement in ambulatory care.

**Design:**

Cross-sectional mixed-mode survey.

**Setting:**

General practitioner and specialist practices.

**Participants:**

Patients aged >18 years seeking care in ambulatory care practices between February and June 2020.

**Methods:**

A 22-item-questionnaire was completed in the practice or at home either on paper or online. Multivariate logistic regression was used to analyse the influence of survey mode and patient characteristics on patient experience with patient safety.

**Results:**

The overall response rate was 71.1%. Most patients completed the questionnaire on site (76.6%) and on paper (96.1%). Between 30.1% to 68.5% of the respondents report the most positive option for patient experience with the main domains of patient safety. A total of 2.9% of patients reported having experienced a patient-safety event (PSE) during the last 12 months. Patients who filled in the questionnaire off site were more likely to report negative experiences for the scales communication & information (OR 1.2, 95% CI 1.0–1.5), rapport & participation (OR 1.4, 95% CI 1.1–1.7) and access (OR 1.3, 95% CI 0.9–1.4) than those who completed it on site. Those who chose a paper questionnaire were more likely to report negative experiences for all five scales compared to web responders.

**Conclusion:**

Routine measurement of patient experience with factors contributing to the occurrence of PSEs can achieve high response rates by offering flexible participation options. Results gained from mixed-mode surveys need to take mode-effects into account when interpreting and using the results. Further research is needed in how to adequately assess number and type of experienced events in routine measurements.

## Introduction

Routine measurement of patient safety from the patients’ perspective is increasingly recognized as an important component of patient safety management systems [1]. While efforts to increase patient safety have long focused on inpatient care, research into patient safety in ambulatory care has been growing over the last years. A systematic review by Panesar et al. [2] indicates that between <1 and 24 patient safety incidents occur per 100 consultations. The included studies were heterogeneous in terms of definition and measurement of patient safety incidents which may partly explain the substantial range. However, when looking only at studies based on patient reports, the variation in observed rates remains considerable with 8 to 45% of participants reporting having experienced a patient safety problem [3–6].

As in inpatient care, it is increasingly acknowledged that involving patients in the safety of their care is an important aspect in systematically identifying safety problems in ambulatory care [7–9]. Patients are in a unique position to provide feedback about their care as they are the only ones who are part of the whole care process [10]. This is even more true in ambulatory care where care is often fragmented, particularly in complex cases when many providers are involved [9]. There is increasing evidence that patients are able to identify factors that correlate with the occurrence of safety incidents [8, 11]. Their reports are reliable [12, 13] and offer unique information that cannot be gathered otherwise [14, 15]. The main safety related areas in ambulatory care refer to medication safety [16], diagnosis [17], coordination of care [18, 19] and communication between health care professionals and communication with patients [20] with overlaps between the areas.

More than one approach will be required to gain a comprehensive picture of patient experience with patient safety. Depending on the measurement goal, a combination of data collection methods may often be useful [21]. However, as with patient experience in general, standardized surveys are likely to remain one of the most important tools, particularly when gaining data suitable for internal and external quality management purposes [22]. For measurement efforts to be worthwhile, the aim needs to be clear and the survey instrument and method tools used need to produce valid, reliable, relevant and manageable results and must be acceptable to the target group [23]. Therefore, instruments should be tested whether they measure what they are intended to measure (validity), produce consistent results under similar circumstances (reliability). They have to be acceptable to users, for example with regard to length, cognitive burden and ease of completion, otherwise the risk of low response is high [24, 25]. Finally, the results obtained need to represent actionable feedback in order to be useable for example for improvement efforts in real-world practice [22].

Various instruments to assess patient experience with patient safety have been developed previously. These have focussed primarily on specific aspects of patient safety or outcomes of safety incidents in ambulatory care such as adverse drug events [26]. In recent years, some instruments have been developed that either measure patients’ experience with actual patient safety events [3, 5], patients’ experience with error-producing or contributing factors [27] or both [28]. The questionnaire developed by Ricci-Cabello et al. [28] consists of 71 items with the majority of items focussing on patients’ experience with safety problems and resulting harm and the remainder of items covering contributing factors in the areas of communication, participation, coordination and access. The researchers found preliminary evidence supporting its reliability and validity. Giles et al. [27] developed a tool measuring patients’ experiences with error-producing factors consisting of 50 items in nine domains (access, communication, external policy, information flow, organisation care planning, patient related factors, physical environment, referrals and task performance). Psychometric testing demonstrated acceptable levels reliability and validity with further development and testing needed. The 16-item-tool developed by Stocks et al. [5, 29] measures patients’ experience with mistakes or problems that could have or have actually worsened their health and could have been prevented. It explicitly encourages free-text responses regarding nature and preventability of the event(s) experienced. Face validity testing showed good results and pilot participants confirmed ease of completion. Geraedts et al. [3] designed a detailed tool recording patient experience with patient safety problems in ambulatory care for use in telephone interviews. It captures the occurrence of seven types of patient safety problems followed by questions on various details such as treatment area, frequency, presumed causes, type and severity of harm, recovery time and others. Suitability of these instruments for routine measurement is limited due to their length, focus, design for use in telephone surveys and/or need for further psychometric testing.

Collecting patient feedback in ambulatory care has long been conducted via paper-based surveys, either on-site or via mail. In recent years collecting feedback electronically also either on site via electronic devices (tablets, kiosk) or via web surveys have gained increasing attention [30].

Compared to on-site surveys mail surveys have the advantage that sampling and distribution can be better controlled [31] but participation rates are generally are lower [32–35]. On-site surveys are associated with a higher burden on practice staff and adherence to distribution protocols may be affected by competing priorities (e.g. higher patient load during influenza season) or process fatigue when questionnaire distribution is intended over a longer time period [31]. Mail surveys tend to have a higher variability of answers [36] and the risk of bias due to socially desirable answers, gratitude effects or effects due to power asymmetries tends to be lower [30–32, 35–37]. Evidence on differences in respondents’ demographic characteristics is mixed, with some authors having found differences in terms of age and gender [33] whereas others did not [31, 36, 37].

Compared to mailed paper surveys web-surveys have the advantage of reduced administration costs, improved timeliness and less data entry errors [24, 38, 39]. However, respondents require Internet access and need to be familiar with use of the internet [24, 40]. Participation rates are usually lower in web surveys [24, 25, 41, 42], however, other survey design factors, such as prenotifications or the number of reminders also play a role [24, 43]. Evidence on differences in response patterns is inconclusive [44, 45] but those having found a difference suggest that the highest differences are found between modes that differed in the degree of personal interaction involved (e.g. self-administered and interview modes) rather than between different self-administered modes (e.g. web and mailed paper surveys) [45, 46]. Web survey participants are likely to be younger [39, 47, 48], more often male [40, 49] and more likely to have a higher educational level [39, 47]. However, although age is still the most consistent predicting factor for participation in web surveys, the proportion of older people responding to online questionnaires has been increasing over the last years [49]. Given these findings, the use of multi-mode survey designs is recommended to provide flexibility, increase response rates and enhance the probability to reach target groups that would be less likely to participate if only one mode was offered [39, 40]. When offered the choice between web and mailed surveys, most participants chose the mail option. Differences in sociodemographic characteristics and response patterns between mail and web respondents in mixed-mode studies were comparable to those found in studies using single method approaches to data collection [46, 47, 50]. We did not find any studies that offered participants a choice between on-site or off-site completion of a questionnaire.

The ASK ME-patient survey was part of a larger project on patients’ perspective of patient safety in ambulatory care. The project followed a threefold aim. The first aim was to develop a questionnaire measuring patient safety events in primary care from the patients’ perspective as well as factors which contribute to their occurrence. The items should reflect the important dimensions of patient safety while being sufficiently generic to be suitable for use in general practitioner as well as specialist practices. The questionnaire should be of reasonable length to support routine use and generate actionable feedback suitable for use in internal risk and quality management. The instrument should further be freely available. The second aim was to gain insight into the current state of patient experience with patient safety in ambulatory care in Germany based on a sufficiently large sample to inform policy makers on potential improvement strategies. The third aim of the project was to develop a mechanism that allows the results to be fed back to the practices in an automated, user-friendly and actionable manner, thereby fostering the systematic involvement of patients in the prevention of adverse events in ambulatory care. This article reports on the results of the patient survey and their implications for patient safety measurement in ambulatory care.

## Materials and methods

For the ASK-ME-study a patient safety event (PSE) was defined as an occurrence (incident, process, procedure or outcome) that increases the risk for an adverse event or that actually leads to an adverse event [51, 52]. A contributing factor is ‘a factor, circumstance or influence that is thought to have played a part in the origin or development, or to increase the risk, of an incident’ [51].

### Instrument

The development and psychometric evaluation of the instrument has been described in detail elsewhere [53]. In short, questionnaire development involved a scoping review of the literature on relevant dimensions and existing instruments, a modified 3-round Delphi-process with 11 experts (four researchers, one member of the German Coalition for Patient Safety, four patients and two clinicians) and cognitive interviews with patients. The resulting questionnaire consisted of 22 items referring to 6 main domains: access, communication, participation, medication safety, coordination of care and experience of PSEs. Nineteen items referred to factors that can contribute to the occurrence of PSEs, three questions referred to the actual experience of a PSE. Response options were on a five-point Likert scale ranging from ‘always’ to ‘never’. For eight questions, a ‘does-not-apply’ answer was provided (see S1 File for the questionnaire). Psychometric evaluation using exploratory and confirmatory factor analysis and internal consistency analysis revealed three composite scales (communication & information, rapport & participation, and medication safety) with good psychometric properties and two single-item scales (access and coordination).

### Study design, setting and participants

A mixed-mode survey design was used to collect cross-sectional data from patients seeking ambulatory care in general practitioner or specialist practices in Germany between February and June 2020. The intended survey period of six weeks had to be extended due to the outbreak of the COVID-19-pandemic. The associated restrictions meant that practices either saw significantly fewer patients, offered only emergency services, kept the time of contact with patients to a minimum or closed completely during March and April 2020.

In Germany, general practitioners (GP) and specialists usually work in their own private practices, patients are free to choose whether to see a GP or a specialist, GPs have no formal gatekeeping function.

Fifty practices should be recruited for the study to achieve a large enough sample for gaining insight into the current state of patient experience with patient safety in ambulatory care. Practices were approached through existing networks and contacts of the research team which was supported by the German Coalition of Patient Safety and the regional Association of Statutory Health Insurance Physicians (Westfalen-Lippe).

For practices to initiate improvement measures based on the survey results, feedback from at least 50 patients is recommended [54]. Sample size calculation was based on an average of 800 patients per practice in the originally intended survey period of six weeks. Assuming a variability of the variables of 70%, a margin of error of 10% and a confidence level of 95%, meant that 74 patients per practice were needed. Further assuming a response rate of 30% meant that 250 questionnaires per practice had to be handed out. Participating practices were provided with the survey materials and received an expense allowance of 200€. A member of the research team was available via telephone for questions throughout the field phase.

All patients aged ≥ 18 years and with sufficient language skills to complete the self-report questionnaire were eligible for inclusion. Practice staff was instructed by the research team to hand out the questionnaire, including cover letter and return envelope, to consecutive patients. They explained the study and informed patients that participation was voluntary, that refraining from taking part was possible at any time and that non-participation would not affect their care. Patients could participate by completing the questionnaire in the practice or at home either on paper or online. This mixed-mode approach was chosen to reduce participation barriers given the heterogeneity of the study population. The URL and access code for the online survey were provided in the cover letter. The access code was identical to the questionnaire-ID to ensure that duplicate questionnaires could be identified in case a patient had completed the questionnaire on paper as well as online. Paper-based questionnaires that were completed on-site were collected in a poll-box that was installed in the waiting room of the practice. For each patient who was given a questionnaire practice staff was asked to collect gender, age, and educational degree on an additional ‘non-responder-page’ that was attached to each questionnaire. This page was collected in the practice and sent to the project team at the end of the data collection period to analyse the non-response bias. Ethical approval was granted by the Ethics Committee of the Medical Association of Physicians (Westfalen-Lippe) and the Faculty of Medicine, University of Münster, Germany.

### Data analysis

Simple frequencies were calculated to describe the sample. Item-based analyses were conducted by calculating the number and percentage of patients answering each of the response categories in each item. The frequencies for the composite scales were calculated by averaging the means of the items within the composite. For cases where more than one item within the composite had a missing value no scale score was calculated. Multivariate logistic regression was used to analyse the influence of survey mode and patient characteristics on patient experience with patient safety using survey mode and demographic characteristics as independent and patient experience as dependent variables. For analysis purposes the outcome variables were dichotomized into patients with positive and negative experiences using the median as cut-off value. To investigate the association between predictors and outcome variables odds ratios were calculated. Results were considered significant at the 5% level. Analyses were carried out using SPSS v26.

### Patient and public involvement

Patients were involved throughout the development process of the questionnaire. Item generation was supported by experts from different fields, including patients and physicians, within a Delphi procedure. To check for comprehensibility and feasibility of the questionnaire patients were involved through cognitive interviews.

## Results

### Sample and response rate

A total of 22 practices were recruited for the study (9 GP practices, 13 specialist practices, see S1 Table for comprehensive list of participating practices). The median number of questionnaires handed out by practices was 228. The main reason for incomplete questionnaire distribution were the restrictions associated with the outbreak of the COVID-19-pandemic. The overall response rate, based on the number of distributed questionnaires, was 71.1%. Most questionnaires were completed on paper (96.1%) and on-site (76.6%). Nearly four out of five paper questionnaires were completed in the waiting room, online questionnaires were somewhat more frequently completed off-site, however, from 16.9% of the online questionnaires, information on the site of completion was not available (Table 1).

**Table 1:** Response rates by survey mode.

	Questionnaires (n)		Response rate	
	Total	median^a^	total	median^b^
**Questionnaires distributed**	4.276	228		
**Questionnaires completed**	**3.042**	139	**71.1%**	63.6%
**Completed^c^ …**				
**… on paper**	2.924	135	96.1%	98.3%
**… online**	118	3	3.9%	1.7%
**Completed^c^ …**				
**… on site**	2.331	97	76.6%	82.0%
**… off site**	691	13	22.7%	18.0%
**… site unknown**	20	2	0.7%	43.1%
**Completed^d^…**				
**… paper on site**	2.287	97	78.2%	83.4%
**… paper off site**	637	12	21.8%	16.6%
**Completed^e^…**				
**… online on site**	44	0	37.3%	0%
**… online off site**	54	2	45.8%	70.8%
**… online site unknown**	20	2	16.9%	43.1%

^a^ median number of questionnaires handed out/completed in participating practices.

^b^ median of response rates in participating practices.

^c^ denominator: all completed questionnaires.

^d^ denominator: all questionnaires completed on paper.

^e^ denominator: all questionnaires completed online.

From the 3042 questionnaires 28 had to be excluded from analyses due to a patient age less than 18 years, leaving feedback from 3014 patients for analysis. Patient characteristics for the whole sample and across survey modes are shown in Table 2. Those who completed the questionnaire in the waiting room tended to be younger, female, to have a higher educational attainment, no chronic disease, and a better subjective health status. Patients who completed the questionnaire on paper were more likely to be 60 years or younger, female, have a lower educational attainment and no chronic disease.

**Table 2:** Patient characteristics across survey modes.

	Participants (n (%))	Participants (n (%))			Participants (n (%))		
	Overall	On site	Off site	χ^2^ (p)	Paper	Online	χ^2^ (p)
**Age**				132.32 (<0.001)			3.27 (0.07)
≤ 60 years	1928 (67.5)	1589 (73.3)	332 (49.6)		1865 (67.8)	63 (59.4)	
> 60 years	928 (32.5)	578 (26.7)	338 (50.4)		885 (32.2)	43 (40.6)	
**Gender**				51.66 (<0.001)			25.61 (<0.001)
Female	1915 (66.9)	1532 (70.7)	378 (55.8)		1862 (67.8)	53 (45.3)	
Male	949 (33.1)	635 (29.3)	299 (44.2)		885 (32.2)	64 (54.7)	
**Educational attainment** ^**a**^				7.26 (0.007)			4.74 (0.03)
Low	793 (27.7)	1542 (71.1)	517 (76.4)		1999 (72.7)	75 (63.6)	
High	2074 (72.3)	628 (28.9)	160 (23.6)		750 (27.3)	43 (36.4)	
**Disease lasting > 3 months**				128.11 (<0.001)			17.46 (<0.001)
No	1624 (56.8)	1070 (49.4)	166 (24.6)		1208 (44.0)	29 (24.6)	
Yes	1237 (43.2)	1097 (50.6)	508 (75.4)		1535 (56.0)	89 (75.4)	
**Subjective Health status**				48.43 (<0.001)			0.23 (0.63)
Good	2070 (71.8)	1642 (75.1)	416 (61.4)		1983 (71.7)	87 (73.7)	
Poor	814 (28.2)	544 (24.9)	262 (38.6)		783 (28.3)	31 (26.3)	

^a^ low educational attainment: below A-level, high educational attainment: A-levels or higher.

**Fig 1:**
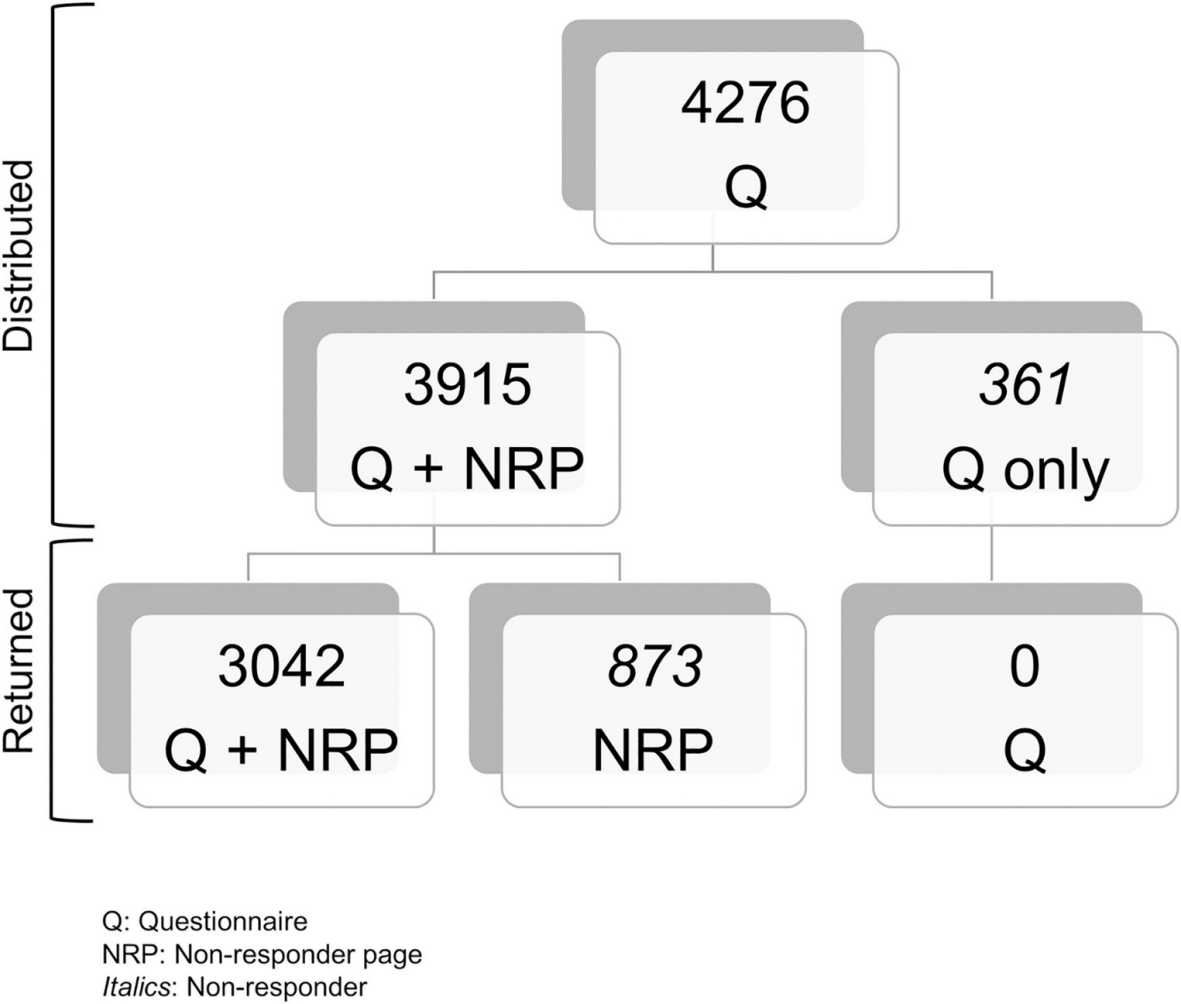
Non-responder.

For 3915 of the 4276 distributed questionnaires practice staff completed the ‘non-responder-page’ (NRP). For all of the 3042 questionnaires returned, a NRP was available. For the 1234 questionnaires that were not returned, a NRP was available for 873 (71%) (Fig 1).

Non-responders were older (56 vs. 51 years, p>0.001) and less likely to have an A-Level or university degree (19% vs. 28%, p<0.001). No difference was found between the two groups in terms of gender (Table 3).

**Table 3:** Demographic characteristics of responders and non-responders.

	Responders	Non-responders	p^a^
	mean (SD)	mean (SD)	
**Age** (years)	51.1 (17.7)	56.1 (17.0)	0,003
	n (%)	n (%)	χ^2^ (p)
**Gender**			5,9 (0,6)
Female	1.927 (67)	483 (64)	
Male	9.665 (33)	268 (36)	
**Educational attainment** ^b^			26,2 (<0,001)
Low	2.094 (72)	159 (17)	
High	793 (28)	689 (81)	

^a^ Student’s t-tests.

^b^ low educational attainment: below A-level, high educational attainment: A-levels or higher.

### Patient experience with contributing factors

Overall, patient experience with the main domains of patient safety is largely positive, between 69.2 and 92.4% report the two positive options (always/often) (Table 4). It could be argued that in the context of patient safety the most positive experience should be aimed at, which is the case for 30.1% to 68.5% of patients.

**Table 4:** Patient experience with contributing factors.

		%^a^				
	Items (n)^b^	Always	Often	Some times	Rarely	Never
Communication & information	6	68.5	23.9	5.2	1.1	0.2
Rapport & participation	4	53.3	29.9	9.9	3.2	1.4
Medication safety	4	60.5	17.9	6.9	3.2	1.8
Coordination of care	1	50.1	19.1	10.8	8.0	12.0
Access	1	30.1	42.0	20.5	6.1	1.3

^a^ Percentages were calculated by averaging the means of the items in each answer category within the composite. For cases where more than one item within the composite had a missing value no scale score was calculated.

^b^ a comprehensive list of all items for the respective factors is provided in S2 Table.

On average, more than two thirds (68.5%) reported that they were always given clear and sufficient explanations and information about their condition and treatment, that they could ask questions and felt taken seriously as measured by the scale communication & information. On average, more than half (53.3%) felt that they were always encouraged to voice worries or sensitive issues, involved in their care and that their provider ensured that explanations had been understood as measured by the scale rapport & participation. Sixty percent of the patients with newly prescribed medicines were always clearly informed about purpose, side-effects, use and application and asked about prescriptions by other providers as measured by the scale medication safety. On average, half of the respondents (50.1%) reported that test results were always available when needed (coordination of care) and for 30.1% it was very easy to get an appointment in urgent cases (access) (Table 4).

### Experience of patient safety events and resulting harm

**Fig 2:**
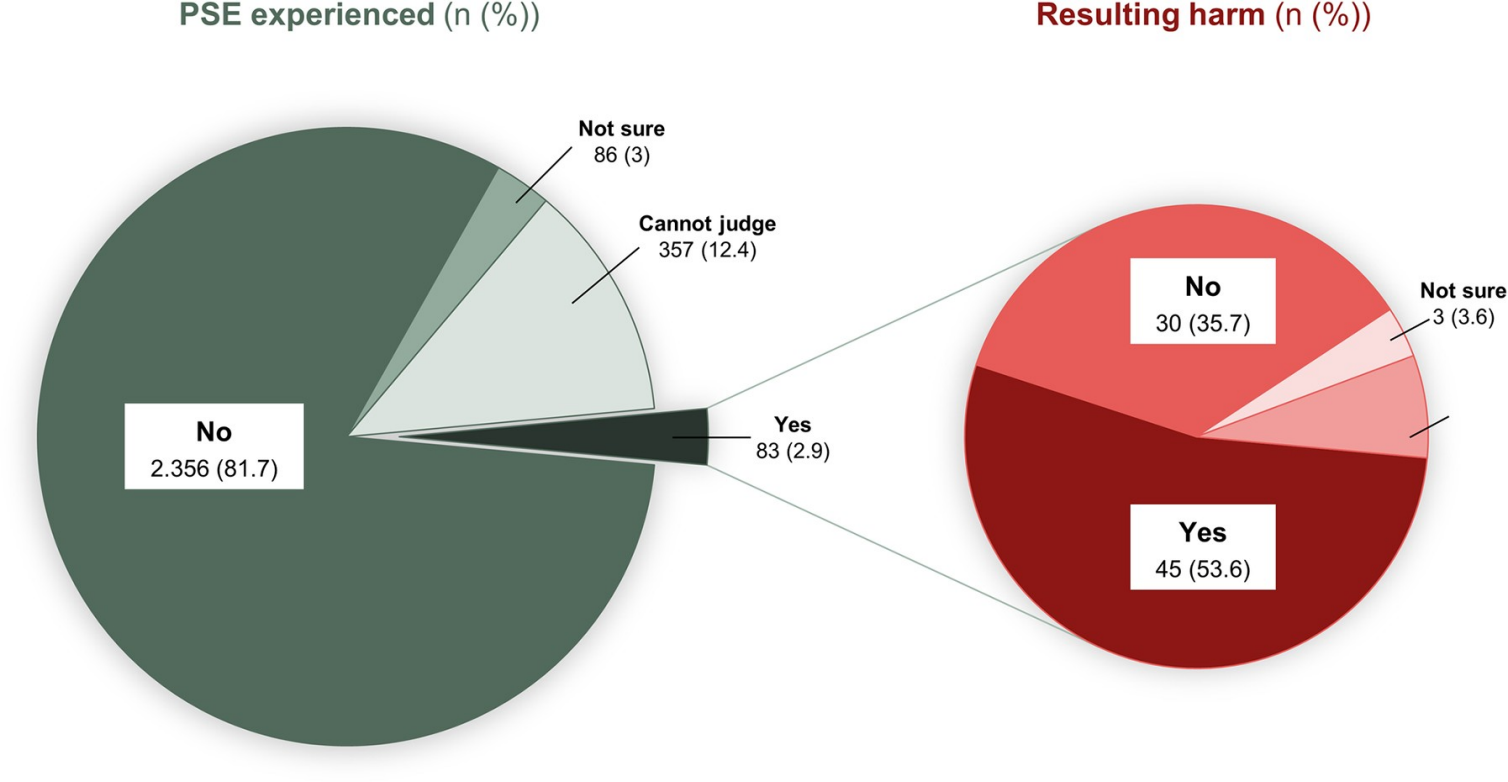
Experience of PSE and resulting harm.

A total of 83 (2.9%) patients reported having experienced a PSE during the last 12 months, 86 (3.0%) were not sure and 357 (12.4%) said that they did not feel in a position to judge whether what they had experienced was a PSE. From those who had experienced a PSE, 45 (54%) reported that it had resulted in physical or emotional harm (Fig 2).

### Effect of survey mode and patient characteristics on patient experience with factors contributing to PSE

Multivariate analysis showed that patients who filled in the questionnaire off site were more likely to report negative experiences for the scales communication & information (OR 1.2, 95% CI 1.0–1.5), rapport & participation (OR 1.4, 95% CI 1.1–1.7) and access (OR 1.3, 95% CI 0.9–1.4) than those who completed it in the waiting room (survey mode 1). Those who chose a paper questionnaire were more likely to report negative experiences for all five scales compared to those who used an online option (survey mode 2). Older age was associated with the scales communication & information (OR 0.6, 95% CI 0.5–0.8), rapport & participation (OR 0.7, 95% CI 0.6–0.8), medication safety (OR 0.7, 95% CI 0.7–0.9), and with coordination of care (OR 1.3, 95% CI 1.0–1.5). Men were more likely to report negative experiences for the scales communication & information (OR 1.2, 95% CI 1.0–1.4) and coordination of care (OR 1.4, 95% CI 1.1–1.6) but more positive experiences for the scale medication safety (OR 0.8, 95% CI 0.7–0.9). A higher level of education was associated with more negative experiences for the scales rapport and participation (OR 1.2, 95% CI 1.0–1.5) and medication safety (OR 1.2, 95% CI 1.0–1.5) and more positive experiences with coordination of care (OR 0.6, 95% CI 0.4–0.7). A poor self-rated health status was associated with more negative experiences on all scales except coordination of care (Table 5).

**Table 5:** Effect of survey mode and patient characteristics on patient experience with factors contributing to PSE.

	Communication & information^a^ (n = 2710)		Rapport & participation^a^ (n = 2705)		Medication safety^a^ (n = 2626)		Access^a^ (n = 2657)		Coordination of care^a^ (n = 2518)	
	OR^b^ (95% CI^c^)	p	OR (95% CI)	p	OR (95% CI)	p	OR (95% CI)	p	OR (95% CI)	p
**Survey mode 1**										
*On-site*										
Off-site	1.2 (1.0–1.5)	0.05	1.4 (1.1–1.7)	0.001	1.0 (0.9–1.2)	n.s	1.3 (1.0–1.6)	0.019	1.1 (0.9–1.4)	n.s
**Survey mode 2**										
*Online*										
Paper	1.9 (1.2–3.1)	0.004	1.8 (1.1–2.8)	0.014	1.7 (1.1–2.7)	0.025	2.0 (1.1–3.7)	0.018	9.4 (3.4–25.9)	<0.001
**Age**										
*≤ 60 years*										
> 60 years	0.6 (0.5–0.8)	<0.001	0.7 (0.6–0.8)	<0.001	0.7 (0.7–0.9)	<0.001	0.9 (0.7–1.1)	n.s	1.3 (1.0–1.5)	0.029
**Gender**										
*Female*										
Male	1.2 (1.0–1.4)	0.02	1.1 (1.0–1.4)	n.s.	0.8 (0.7–0.9)	0.008	0.9 (0.8–1.1)	n.s	1.4 (1.1–1.6)	0.001
**Education**										
*Low*										
High	1.1 (0.9–1.3)	n.s.	1.2 (1.0–1.5)	0.012	1.2 (1.0–1.5)	0.014	0.9 (0.7–1.1)	n.s	0.6 (0.4–0.7)	<0.001
**Chronic disease**										
*No*										
Yes	0.8 (0.7–1.0)	n.s.	0.8 (0.8–1.0)	n.s	1.0 (0.8–1.1)	n.s	1.0 (0.8–1.2)	n.s	0.9 (0.7–1.1)	n.s
**Subjective health status**										
*Good*										
Poor	1.5 (1.2–1.8)	<0.001	1.5 (1.2–1.8)	0.012	1.3 (1.1–1.6)	0.006	1.5 (1.3–1.9)	<0.001	0.6 (0.4–0.7)	n.s

^a^ reference category: negative experience.

^b^ Odds ratio.

^c^ Confidence interval.

*Italics*: reference category.

n.s.: not significant.

## Discussion

This study reports on the results of a large, mixed-mode survey of patient experiences with patient safety in ambulatory care and their implications for routine patient safety measurement from the patient perspective. The overall response-rate was very high, with most patients choosing to complete a paper questionnaire in the practice. Patients’ experience with factors contributing to the occurrence of PSE was mainly positive, the most frequently reported problems referred to access and coordination, and aspects of the doctor-patient interaction that do not reflect traditional behavioural patterns. Only a small proportion of patients reported having experienced a PSE in the last 12 months.

### Routine measurement of patient safety

The excellent response rate of this study indicates a high willingness of patients to provide feedback on their care if the response burden is acceptable, one factor for this being the length of the questionnaire [55]. Other surveys of patient experience with patient safety in ambulatory care using longer questionnaires yielded response rates of 12%, 18% and 64% in a telephone survey [3], a mailed survey [4] and a waiting room survey [6] respectively. Giles et al. [27] point out that conducting on site surveys with long questionnaires face the challenge that the time to complete and return the questionnaire maybe longer than the waiting time and that completion may be interrupted by being called in to the doctor. Low response rates can prevent providers from using the results due to lack of trust in their validity [30]. Furthermore, the amount of data generated needs to be manageable, otherwise it is more likely that it will not be used for improvement efforts by practice staff [30, 56].

Another reason for the high response rate in this study may be the offer of flexibility regarding the timing of questionnaire completion and that patient subgroups could be accessed that may otherwise not have participated [39]. The choice between the paper and online option did not markedly increase survey participation, a result that has also been found in other studies [57]. However, since all patients were given a paper questionnaire with access information for the online option provided in the cover letter, it is conceivable that completing the paper questionnaire was perceived as the easiest option. This is supported by the literature which suggests that participation rates seem to be mainly influenced by ease of access and familiarity with the method [48]. International initiatives on routine collection of patient-reported experience measures have also adopted multi-mode administration to support high participation [1].

Survey mode was an independently associated with patient experience in this study, but the odds ratios were low for all predictors. Patients who completed the questionnaire on site reported more positive experiences with rapport and participation, communication and information and access compared to those who completed it off site. This is in line with existing evidence that suggests that patients are more reluctant to report on negative experiences in the presence of staff, particularly to ‘staff-related’ aspects of care. Patients who complete the questionnaire at a later time may have had more time to reflect on the consultation and its effectiveness and may feel more free to be critical due to perceived anonymity [31, 36]. Patients who chose the web survey option reported more positive experiences for all five scales. However, these results need to be interpreted with caution due to the small number of patients in the online group. In line with the literature, age and subjective health status were the most consistent predictors of experience [58], gender and education were predictive for some but not for all scales which is also in line with the literature where there is inconclusive evidence about these characteristics [59].

### Patient experience with contributing factors

This study reports on the results of a large, mixed-mode survey of patient experiences with patient safety in ambulatory care and their implications for routine patient safety measurement from the patient perspective. The results indicate that most patients report a predominantly positive experience, particularly regarding communication and information in general or information on medication. This positivity tendency is in line with other research looking at patient experience with factors contributing to the occurrence of PSE [4, 27] as well as patient experience measurements in ambulatory care in general [60, 61]. Reasons for this tendency are complex involving aspects such as the actual quality of care but also loyalty and gratitude effects, social desirability bias, power asymmetries, cognitive dissonance effects and patient characteristics [62]. Given this complexity, it is recommended that the focus should not be on the absolute scores of the patient reports as an indicator for good (or poor) quality of care but on the relative scores, thereby using them as a management tool for identifying improvement areas [63]. Experiences with access and coordination of care are less positive. This also applies for those aspects of the patient-doctor-interaction that require behaviours or actions which do not reflect the traditional roles of patients and/or doctors. These findings are supported by other studies indicating that in the often fragmented setting of ambulatory care, patients report coordination and access as areas in need of improvement [64, 65] and by studies indicating that concepts such as Shared Decision Making are not yet as normal a part of care as would be desirable [66].

### Patient experience with errors

The proportion of 2.9% of patients having experienced a PSE within the last 12 months is considerably lower than those in other studies, which identified prevalence rates between 7.9% and 16.0% [3, 5, 6], Ricci-Cabello even identified a rate of 45% [4]. This discrepancy may be partly due to methodological reasons. One study conducted computer-assisted telephone interviews which allow to a certain extent for clarifying question meanings and other queries. Furthermore, patients in this study received a short introductory explanation on the concept of error from the interviewer and were then asked about errors in seven medical areas with various examples given for errors in each area [3]. This may have triggered experiences patients would not have judged themselves as being a PSE when asked in a more general way. In a population-based study, citizens aged ≥ 15 years were asked in standardized personal interviews whether they had experienced at least one potentially harmful preventable problem in any primary care setting in the last 12 months. The term problem may have triggered other (and possibly more) incidents than when the term error would have been used. Furthermore, when participants answered ‘no’ to the general question, a follow-up question giving a list of examples of possible errors inquired if one the these examples had happened to the interviewee [5]. A study applying a self-administered questionnaire to be completed in the waiting room asked patients whether a doctor had ever made a mistake in their care. The authors state that the term mistake was deliberately chosen over the term medical error because evidence indicates patients’ confusion about the latter term [6]. However, it is still conceivable that incidents triggered in patients’ memories by the term mistake are different from those triggered by other terms such as problem or safety event. In a mailed survey patients of primary care practices were asked whether they believed they had any problems related to one or more of 12 listed problems, including examples such as problems related to appointments or problems related to communication and coordination between health care professionals [4]. The broad wording may have resulted in a higher prevalence rate than a question asking about more formal concepts such as errors, mistakes, or possible harmful preventable problems. All four patient surveys offered patients a response option indicating that they were not sure or did not know whether they had experienced a PSE, however none of the studies reported the proportion of patients using this option. While several studies explored patients’ conceptualization of patient safety [67, 68], the number and types of events triggered by different wordings when asking about PSE and the effect of providing introductory explanations and examples as well as the choice of examples on prevalence rates have not yet been systematically explored.

### Strengths and limitations of this study

A strength of this study is the large sample and the high response rate which support the internal validity of the results. Furthermore, the instrument used demonstrated good psychometric properties and underwent a rigorous development process with patients being involved throughout the process of drafting and consenting content as well as testing the instruments’ face validity. However, the self-selection of the practices bears the risk that the results may provide an overoptimistic picture. It is conceivable that participating practices are more motivated to engage in patient safety and may already have specific safety interventions or systems in place. The self-selection of patients may also have introduced bias with non-responders in this study being older and less likely to have an A-level or university degree which has been demonstrated to be associated with patient experience in some studies [63]. With the questionnaire being distributed in the practice, only those patients who visited the practice within the survey period (which also included the restrictions due to the outbreak of the COVID-19 pandemic) were included. The self-administration of the questionnaire which was only available in German meant that patients who had sufficient reading, writing and German language skills were more likely to participate. Language barriers are considered a risk factor for safe patient care [69]. The low proportion of patients choosing the online survey means that results regarding this survey mode need to be interpreted with caution.

## Conclusion

Patients’ reports of patient safety in ambulatory care are an important component of patient safety improvement efforts. Routine measurement of patient experience with factors contributing to the occurrence of PSEs can achieve high response rates by offering flexible participation options and using an instrument that is of reasonable length. Involving patients in the process of developing and testing the questionnaire supports acceptability and practicability of the questionnaire. Results gained from mixed-mode surveys need to take mode-effects into account when interpreting and using the results.

More research is needed on how to assess patient experience with the occurrence of PSEs in standardized routine measurements since question wording and content as well as survey-mode may affect number and type of reported events. This should include international comparative studies to assess the robustness of the instrument in different health system contexts and languages.

## Supporting information

S1 File ASK ME questionnaire. (DOCX)

S1 Table Characteristics of participating practices. (DOCX)

S2 Table Patient experience with contributing factors. (DOCX)

S1 Dataset (XLSX)
