# Supplementary figures and images for: ASK ME!—Routine measurement of patient experience with patient safety in ambulatory care: A mixed-mode survey
Source: PLoS One. 2021 Dec 1;16(12):e0259252. doi: 10.1371/journal.pone.0259252 (PMC8635405; doi:10.1371/journal.pone.0259252)

**S1 ASK ME questionnaire, original (German) version**


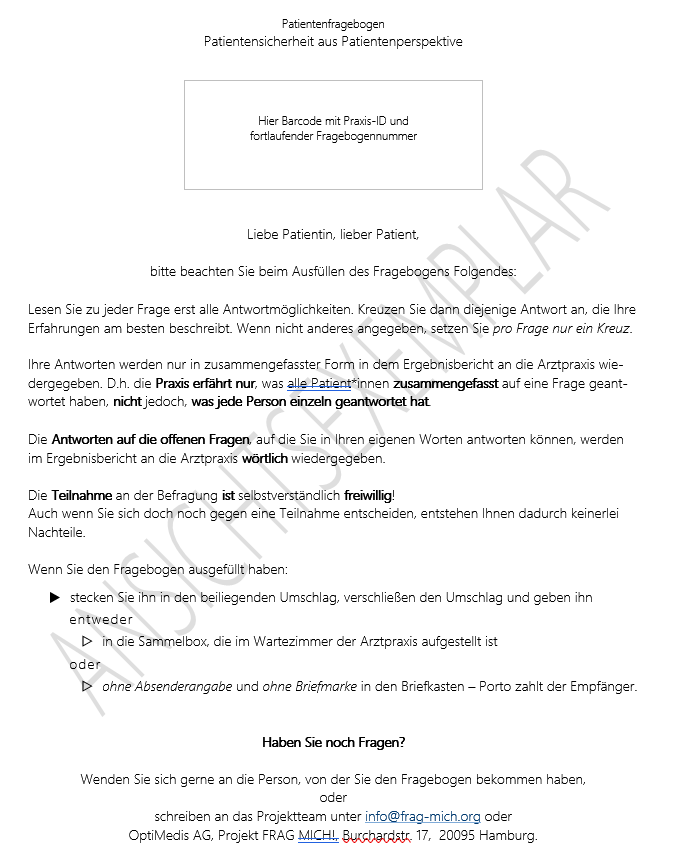


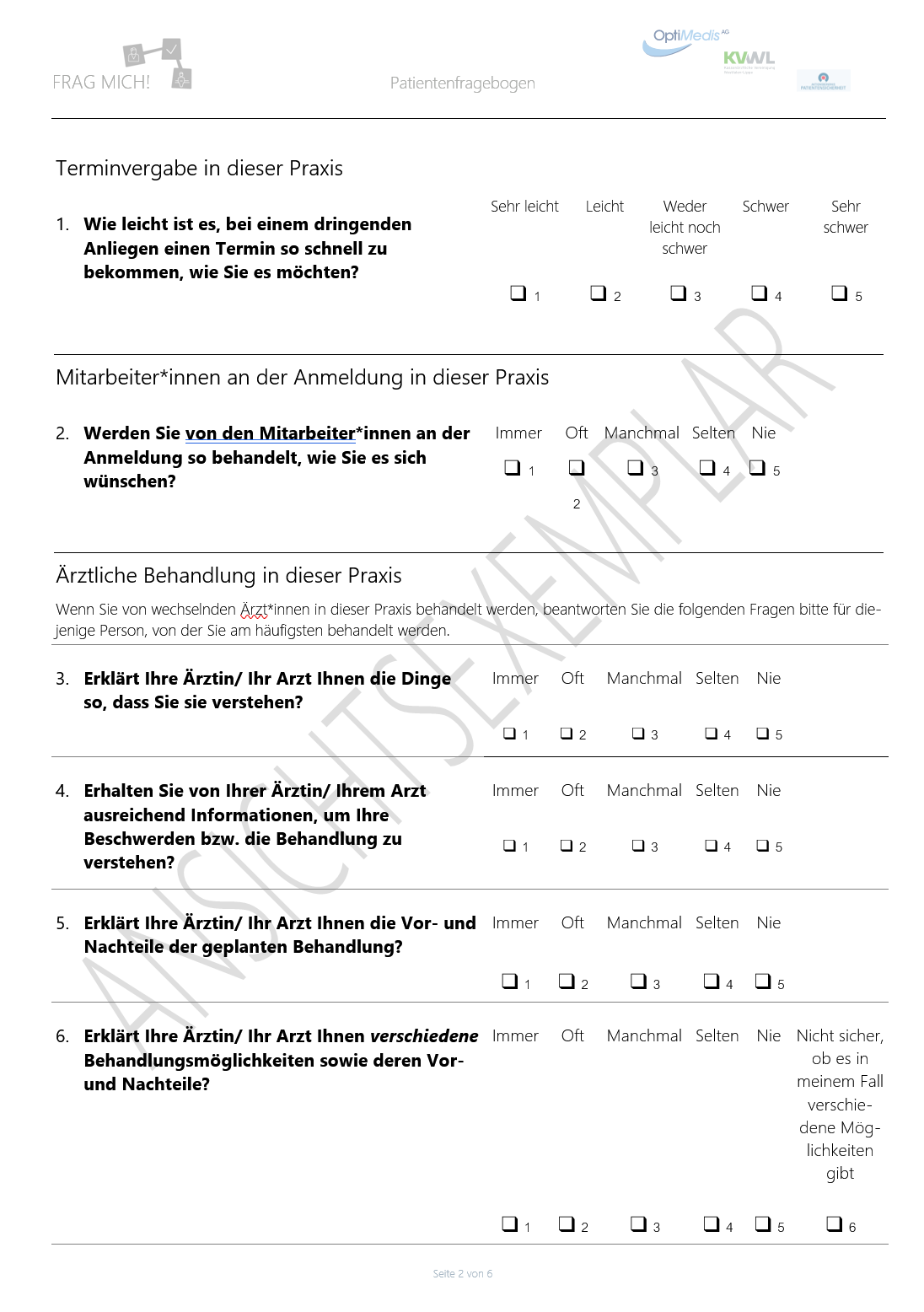


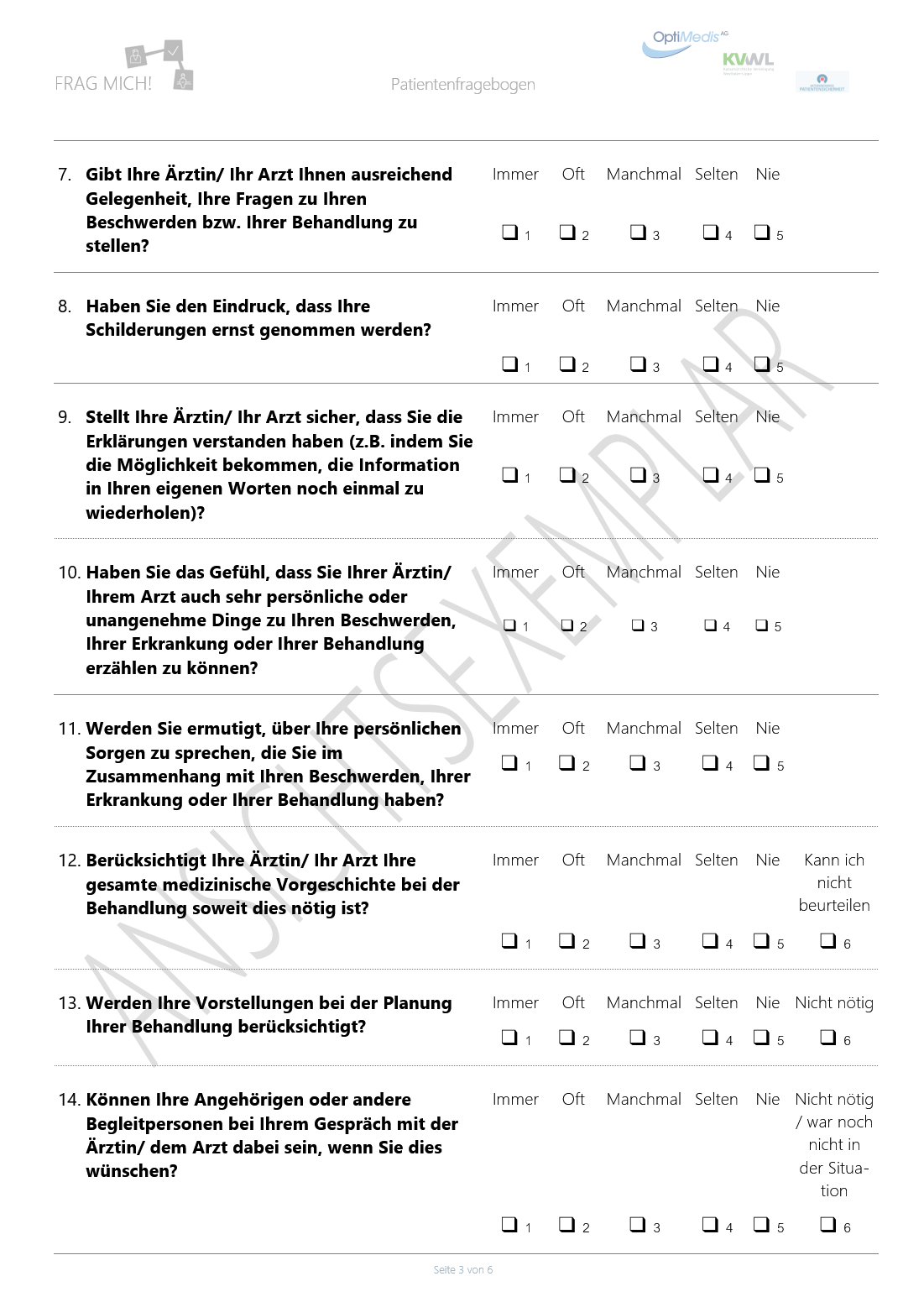


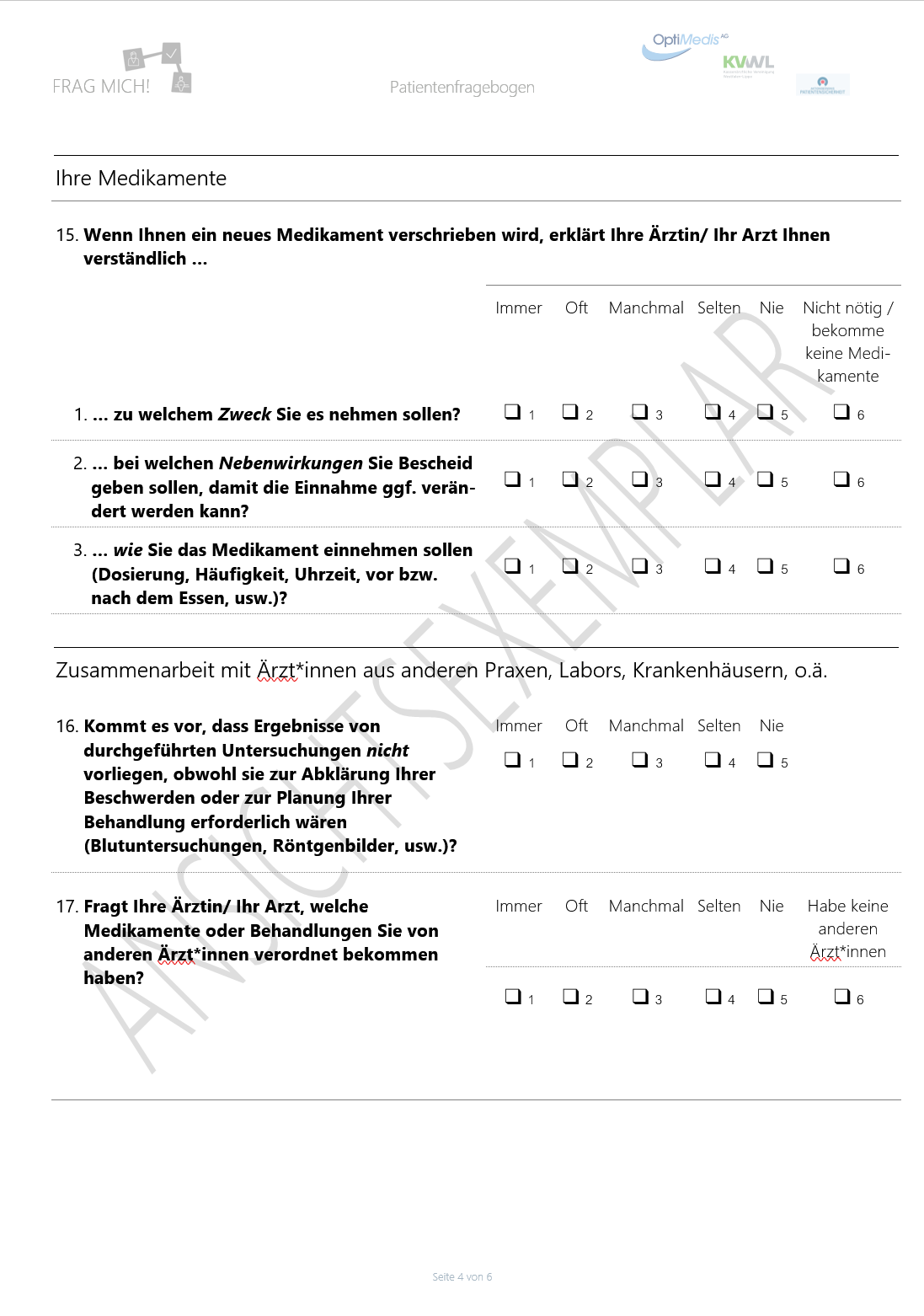


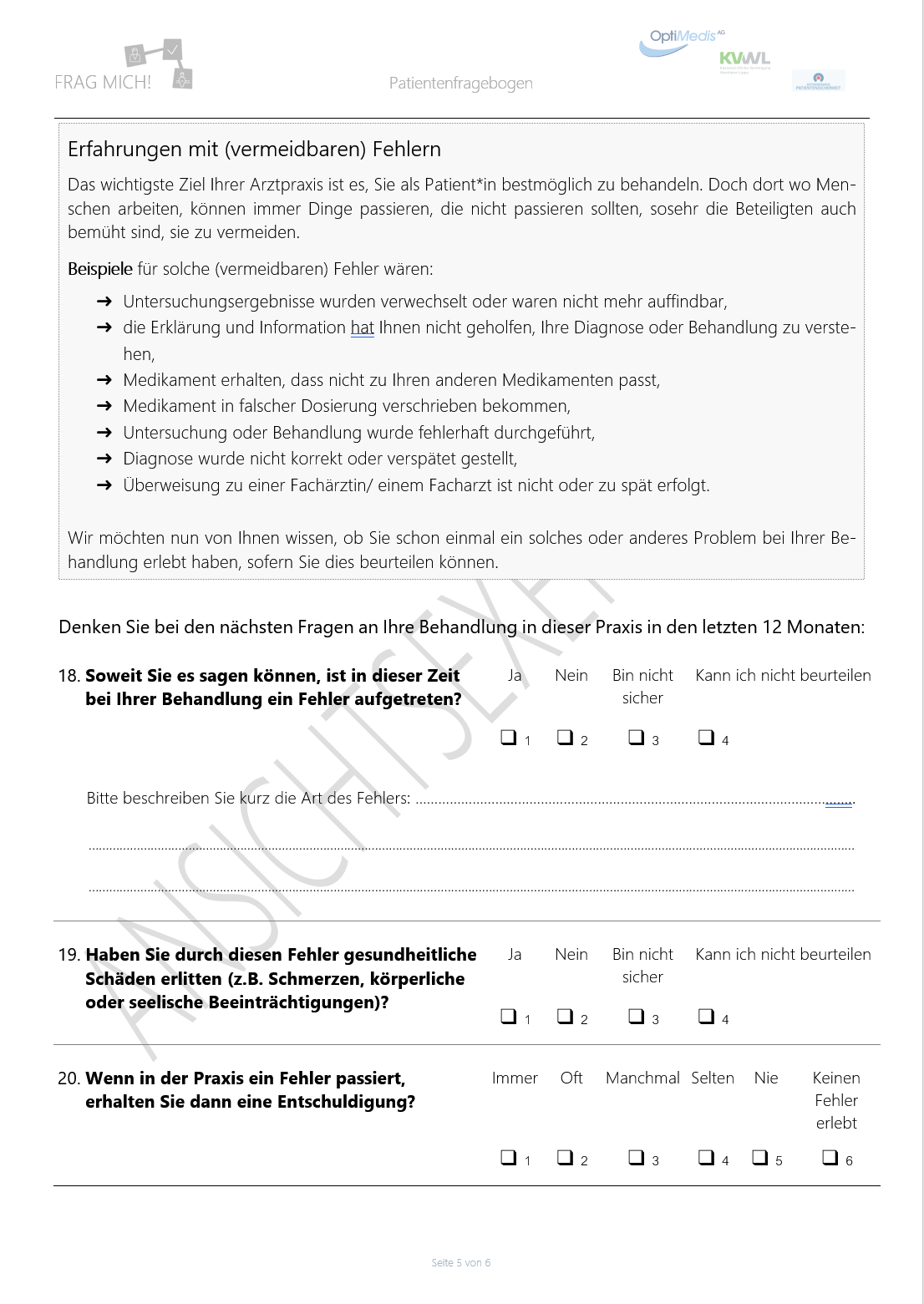


**S1 ASK ME questionnaire, English version**


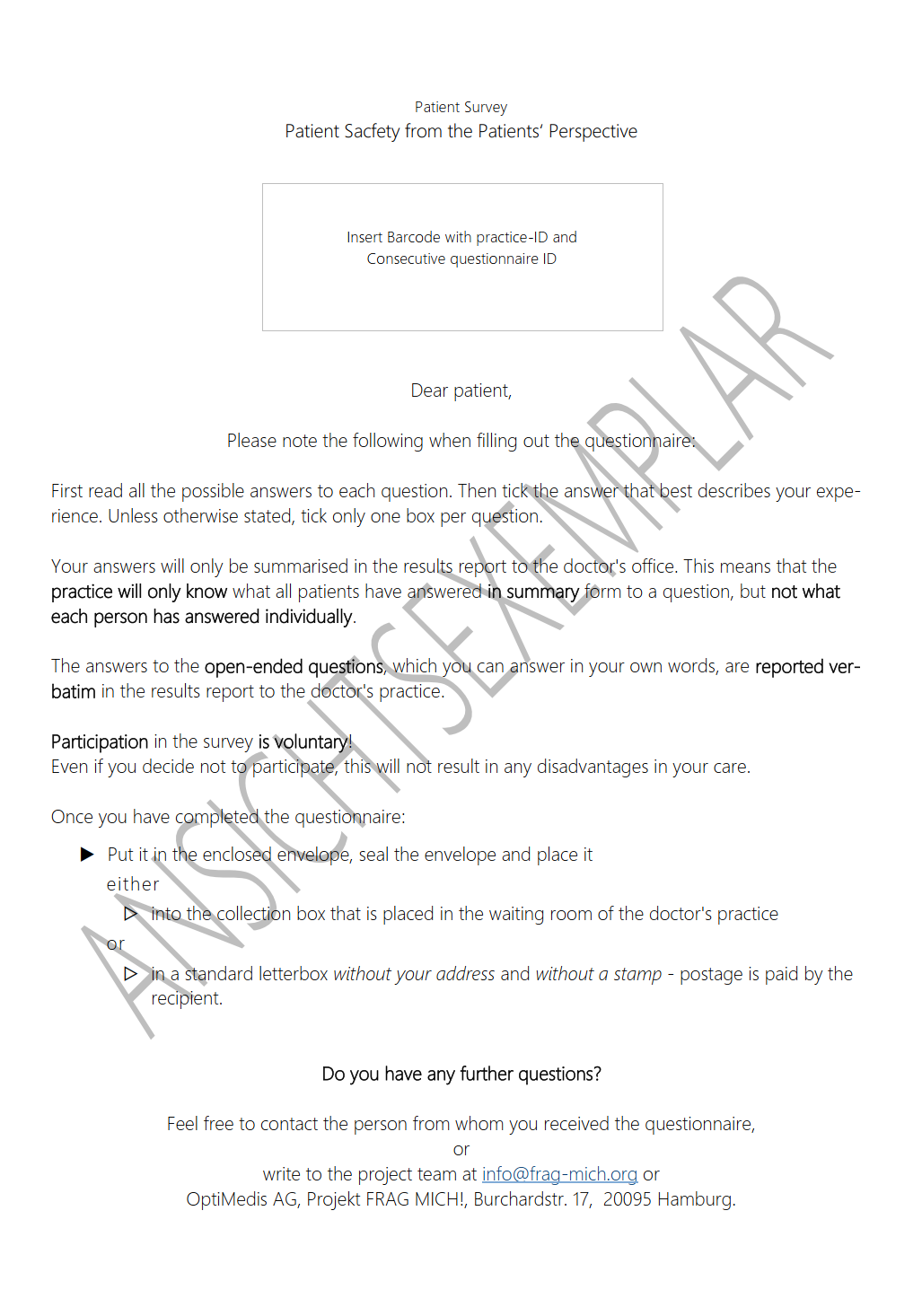


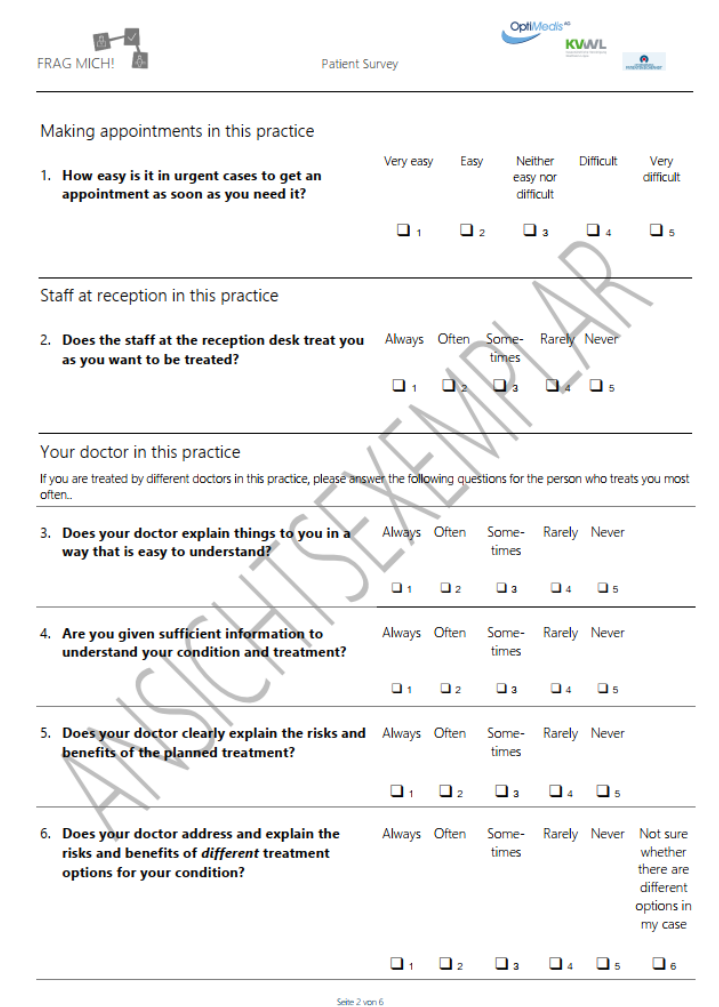


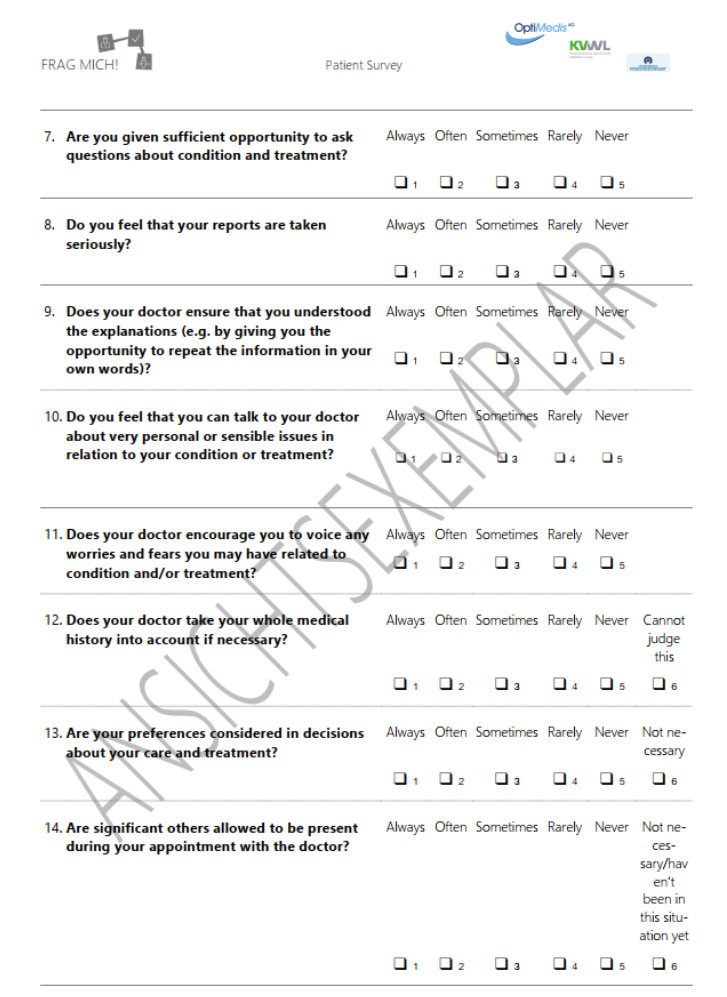


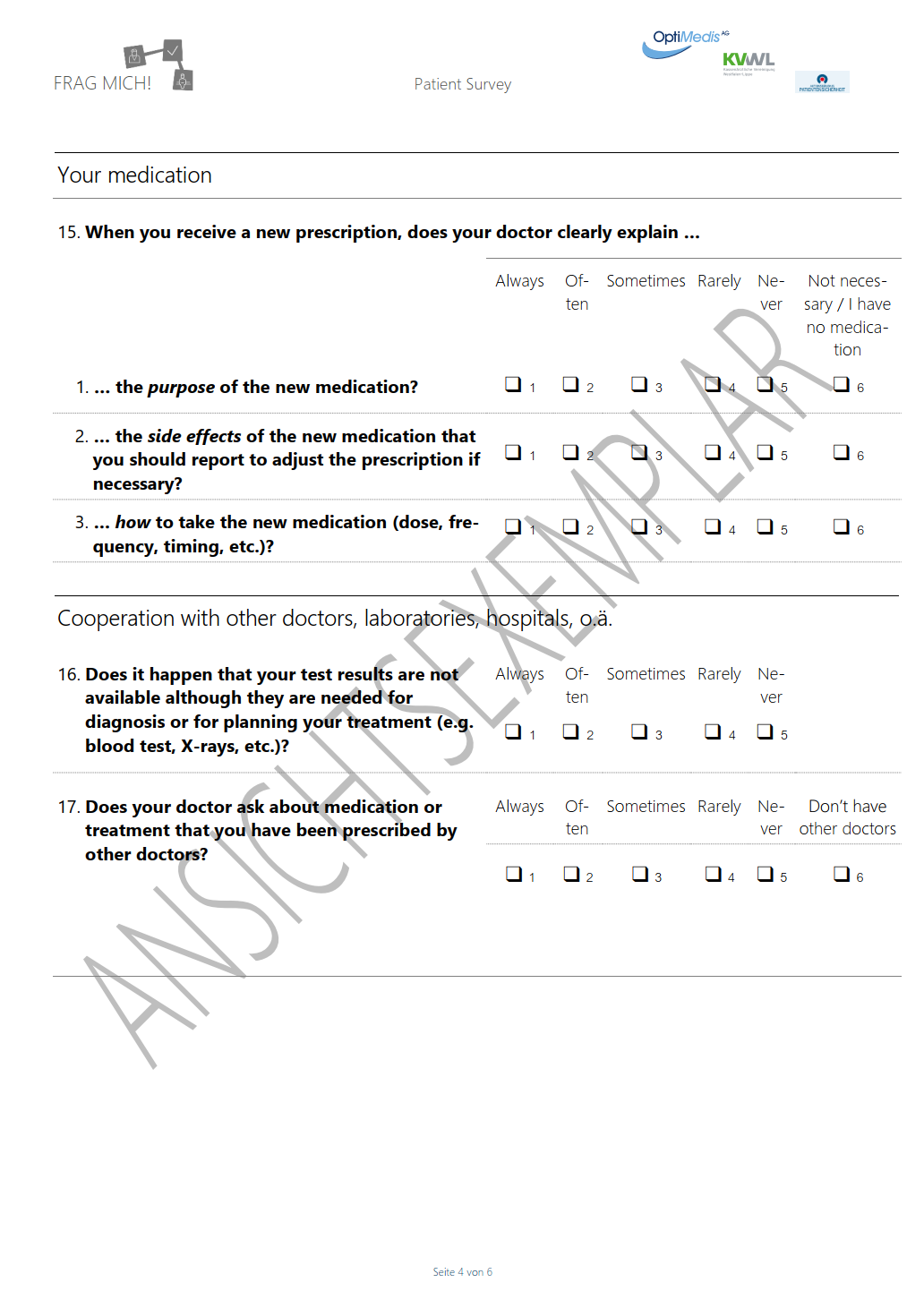


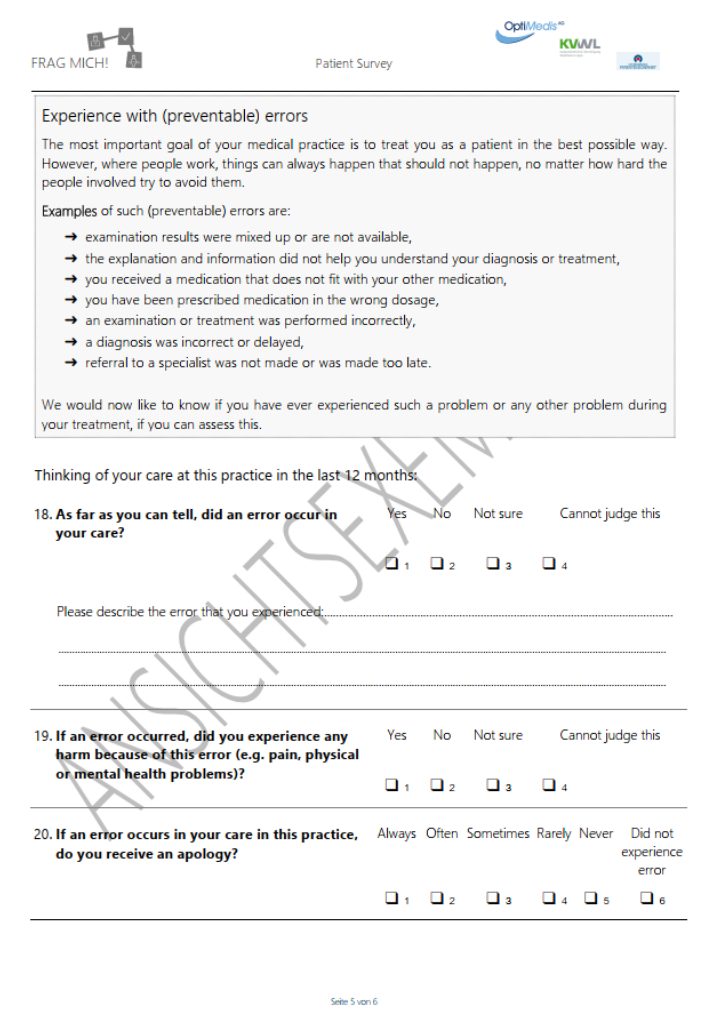


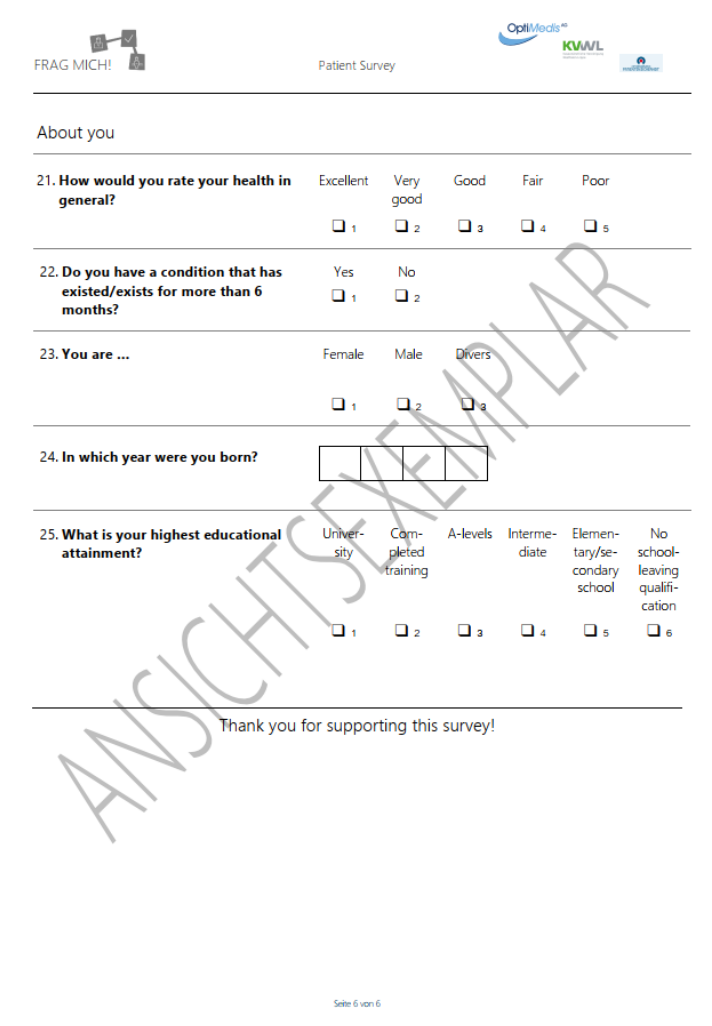

Supplement: S1 File — (DOCX) [file pone.0259252.s001.docx]
