# Supplementary material for: ASK ME!—Routine measurement of patient experience with patient safety in ambulatory care: A mixed-mode survey
Source: PLoS One. 2021 Dec 1;16(12):e0259252. doi: 10.1371/journal.pone.0259252 (PMC8635405; doi:10.1371/journal.pone.0259252)
Supplement: S1 Table — (DOCX) [file pone.0259252.s002.docx]

# S2 Participating practices

**S2 Table 1. Characteristics of participating practices**

|  | Participating practices  n | participating patients per practice  n (%) |
| --- | --- | --- |
| **General practitioner practice** | **9** | **1015 (33.4)** |
| **Specialist practice,** of these  Obstetrics & Gynecology  ENT  Surgical practice  Cardiology  Internal Medicine and diabetology  Neurology, psychiatry & psychotherapy  Ophtalmology  Gastroenterology und Oncology | **13**  3  2  2  2  1  1  1  1 | **2027 (66.6)**  654 (21.5)  472 (15.5)  356 (11.7)  210 (6.9)  140 (4.6)  135 (4.4)  45 (1.5)  15 (0.5) |
