# Supplementary material for: ASK ME!—Routine measurement of patient experience with patient safety in ambulatory care: A mixed-mode survey
Source: PLoS One. 2021 Dec 1;16(12):e0259252. doi: 10.1371/journal.pone.0259252 (PMC8635405; doi:10.1371/journal.pone.0259252)
Supplement: S2 Table — (DOCX) [file pone.0259252.s003.docx]

# S3 Appendix Patient experience with contributing factors

**S3 Table 1. Scale Communication & Information**

|  | % | | | | |
| --- | --- | --- | --- | --- | --- |
|  | Always | Often | Sometimes | Rarely | Never |
| **Communication & Information**^a^ | **68.5** | **23.9** | **5.2** | **1.1** | **0.2** |
| Clear explanation of things | 74.9 | 20.8 | 3.6 | 0.6 | 0.1 |
| Sufficient information to understand condition and treatment | 69.5 | 25.5 | 4.1 | 0.8 | 0.1 |
| Sufficient opportunity to ask questions about condition and treatment | 71.6 | 22.4 | 4.9 | 0.9 | 0.2 |
| Patients felt taken seriously | 71.8 | 22.2 | 4.9 | 1.1 | 0.1 |
| Risks and benefits of planned treatment clearly explained | 67.3 | 24.9 | 6.0 | 1.5 | 0.3 |
| Different treatment options addressed, and risks and benefits addressed and explained | 59.7 | 29.6 | 8.1 | 1.9 | 0.7 |

^a^Percentages were calculated by averaging the means of the items in each answer category within the composite. For cases where more than one item within the composite had a missing value no scale score was calculated.

**S3 Table 2. Scale Rapport & Participation**

|  | % | | | | |
| --- | --- | --- | --- | --- | --- |
|  | Always | Often | Sometimes | Rarely | Never |
| **Rapport & Participation**^a^ | **53.3** | **29.9** | **9.9** | **3.2** | **1.4** |
| Patient preferences are taken into account | 54.4 | 35.8 | 7.7 | 1.7 | 0.4 |
| Encouraged to voice worries and fears related to condition and/or treatment | 47.1 | 31.6 | 13.4 | 5.2 | 2.6 |
| Doctor ensures understanding of explanations | 51.5 | 32.0 | 10.8 | 4.2 | 1.4 |
| Patients feel they can address very personal or sensitive issues in relation to their condition or treatment | 65.1 | 23.5 | 8.3 | 2.0 | 1.1 |

^a^Percentages were calculated by averaging the means of the items in each answer category within the composite. For cases where more than one item within the composite had a missing value no scale score was calculated.

**S3 Table 3. Scale Medication Safety**

|  | % | | | | |
| --- | --- | --- | --- | --- | --- |
|  | Always | Often | Sometimes | Rarely | Never |
| **Medication Safety**^a^ | **60.5** | **17.9** | **6.9** | **3.2** | **1.8** |
| Clear explanation of purpose of newly prescribed medication | 80.3 | 15.3 | 2.9 | 1.1 | 0.3 |
| Clear explanation of side effects of newly prescribed medication that should be reported to make adjustments if necessary | 77.8 | 15.9 | 4.0 | 1.6 | 0.7 |
| Clear explanation of how to take newly prescribed medication (dose. frequency. timing. etc.) | 57.4 | 25.0 | 10.6 | 4.7 | 2.3 |
| Patients are asked about medication or treatment prescribed by other doctors | 51.8 | 23.3 | 13.3 | 6.9 | 4.6 |

^a^Percentages were calculated by averaging the means of the items in each answer category within the composite. For cases where more than one item within the composite had a missing value no scale score was calculated.

**S3 Table 4. Access**

|  | % | | | | |
| --- | --- | --- | --- | --- | --- |
|  | Always | Often | Sometimes | Rarely | Never |
| Ease of getting appointment in urgent cases | 30.1 | 42.0 | 20.5 | 6.1 | 1.3 |

**S3 Table 5. Coordination**

|  | % | | | | |
| --- | --- | --- | --- | --- | --- |
|  | Always | Often | Sometimes | Rarely | Never |
| Test results not available when needed | 50.1 | 19.1 | 10.8 | 8.0 | 12.0 |
